# Stigmatizing Attitudes toward People Living with HIV among Young Women Migrant Workers in Vietnam

**DOI:** 10.3390/ijerph19116366

**Published:** 2022-05-24

**Authors:** Toan Ha, David Givens, Trang Nguyen, Nam Nguyen

**Affiliations:** 1 Department of Infectious Diseases and Microbiology, School of Public Health, University of Pittsburgh, Pittsburgh, PA 15261, USA; dlg43@pitt.edu; 2 Institute of Social and Medical Studies, Hanoi 100000, Vietnam; nttrang@isms.org.vn (T.N.); ntnam@isms.org.vn (N.N.)

**Keywords:** HIV, stigmatizing attitudes, women migrant workers, industrial zones, Vietnam

## Abstract

Despite intensive HIV education and prevention efforts in the past few years, stigmatizing attitudes toward people living with HIV (PLWH) remain a major barrier to HIV prevention and treatment efforts in Vietnam. The purpose of this study was to examine the prevalence of stigmatizing attitudes regarding HIV and identify correlative factors that impact the perceptions of PLWH among a heretofore overlooked demographic in Vietnamese society: women who are migrant workers in designated industrial zones (IZs). A cross-sectional study was conducted among 1061 women migrant workers aged 18 to 29 from January 2020 to November 2020 in Hanoi, Vietnam. Stigmatizing attitudes toward PLWH were measured using a four-item scale. Multiple logistic regression was conducted to examine the factors associated with stigmatizing attitudes. Our findings indicate both substantial levels of stigma persisting among this demographic group as well as the influence of important mitigating factors on the expression of HIV-related stigma. Over seventy-six percent (76.2%) of the participants reported having at least one of the four stigmatizing attitudes. Greater levels of stigmatizing attitudes toward PLWH were significantly associated with lower HIV knowledge, lower levels of education, and identifying as Kinh (the ethnic majority in Vietnam). Additionally, this study found that questions framing HIV infection through a familial lens were significantly associated with lower rates of stigmatizing responses. The high overall levels of stigmatizing attitudes toward PLWH among the study participants suggests that there is an urgent need for the development of culturally appropriate interventions and outreach education activities to reduce stigmatizing attitudes toward PLWH among women who are migrant workers working in the IZs in Vietnam. This study adds to both the existing literature and current efforts and policies around HIV in Vietnam by empirically suggesting that familial-based messaging may be a powerful potential narrative for interventions addressing HIV-related issues such as stigma.

## 1. Introduction

Despite over three decades of progress, HIV continues to be a major global public health issue. Advances along all aspects of the HIV care continuum, including HIV prevention strategies, testing, linkage to care, antiretroviral therapy (ART) treatments, and retention in care, have transformed HIV from a fatal pandemic into a chronic, manageable health condition; however, the persistence of both socio-behavioral barriers and biomedical resource limitations contribute to HIV’s ongoing ignominy as a leading cause of death worldwide [1,2]. Globally, in 2020 alone, an estimated 1.5 million people became newly infected with HIV, raising the current estimated total number of people who are living with HIV (PLWH) to 37.7 million [3]; an estimated 36.3 million people have died because of the epidemic [3]. The vast majority of PLWH are currently living in low- and middle-income countries (LMICs). As of 2020, this includes an estimated 20.7 million (54%) in sub-Saharan Africa, 5.8 million in Asia and the Pacific (15%), 4.9 million (13%) in Western and Central Africa, and 2.2 million (6%) in Western and Central Europe and North America [4]. What is more, an estimated 27% of all PLWH in 2020 did not have access to HIV treatment, and 16% of them did not know their HIV status [3]. In many countries, key at-risk populations, including sex workers, men who have sex with men, people who inject drugs, transgender people, and vulnerable migrant populations, continue to experience higher HIV rates of incidence and AIDS-related mortality than their countries’ general populations [3]. Where data are available, HIV-related stigma and discrimination have been determined to be one of the key barriers to HIV testing, treatment, and care services across the globe, especially in LMICs [5,6]. Indeed, the preponderance of evidence suggests that across cultures, HIV-related stigmas impede every step along the HIV continuum of care, particularly the utilization of HIV testing, linkage to and engagement in care, and medication adherence [7,8].

Stigma can be understood as a process in which classes or groups of people are negatively stereotyped and labeled as socially undesirable by others with greater power and influence [9]. One particularly powerful form of stigma stems from a society’s associations with specific diseases. While a growing number of studies are addressing HIV-related stigma in other LMICs [5,6,10,11], only a few have been conducted among the general population in Vietnam. Vietnam experiences significant HIV burdens, and HIV transmission and access to the care continuum remain major public health concerns in the country. Today, there are an estimated 250,000 people living with HIV (PLWH) in Vietnam, including disproportionate burdens among key populations that include sex workers, men who have sex with men (MSM), and intravenous drug users [12]. The fact that HIV in Vietnam remains highly stigmatized complicates efforts to prevent HIV transmission and engage at-risk communities [13,14]. Indeed, intersecting identities can create compounding layers of stigma within key populations impacted by HIV (i.e., sexual orientation, gender identity, sex work, and illicit drug use) that reify barriers to HIV testing and treatment in Vietnam [15]. Stigma-driven barriers not only harm individuals and communities but also threaten to keep Vietnam from achieving the Joint United Nations Program on HIV/AIDS (UNAIDS)’s target of zero new AIDS cases by 2030 [16].

Perceptions of stigma are informed by cultural expectations [17], and experiences thereof may vary considerably from culture to culture (e.g., in collectivist rather than individualist cultures) [18]. Studies examining the relationships between culture and HIV-related stigma document that PLWH in collectivistic societies are more likely to experience stigma than those in individualistic societies [19]. This may well be the case in Vietnamese collectivist culture as well. Yoshioka and Schustack (2001) described the tendencies of some southeast Asian collectivist cultures to define and enforce social expectations against “immoral” or “deviant” behaviors, including through the use of social myths about risks and social consequences [20]. The perpetuation of these social narratives is a key social mechanism that perpetuates HIV-related stigmatization [20].

During early efforts to raise awareness of HIV prevention in Vietnam, HIV was often portrayed using negative and frightening images and posters. Posters that proclaimed “HIV equals death,” for example, were specifically designed to scare people into adopting the desired avoidance behaviors [21,22]. Negative associations with HIV were further compounded by its association with injection drug users and sex workers, who were already stigmatized in much of Vietnamese society as “social evils” [23]. This approach to early public health HIV campaigns has had serious and long-term consequences. In the eyes of many in Vietnamese society today, PLWH are still often assumed to have engaged in culturally stigmatized “immoral” behaviors, with the further implication being that they “deserve” their HIV diagnosis [23]. Vietnam has worked in recent years to reduce HIV-related stigma toward PLWH, including through the implementation of a rights-based approach to HIV prevention [24]. Vietnam was also the first Southeast Asian country to commit to achieving the 90-90-90 global HIV targets by 2020 [25]. Despite these efforts, recent studies show that stigma toward PLWH still remains prevalent in Vietnam [15,26]. PLWH continue to report experiencing stigmatization due to internalized shame, negative social perceptions and judgments, and discrimination at both the community and family level [27]. Specifically, PLWH in Vietnam still often experience interpersonal social avoidance, perceived anger, and social rejection [27], and report having experienced stigma and discrimination in healthcare settings, at ART clinics [13,14], and in family and community settings [27].

Since Vietnam implemented an “open door” economic policy in 1986, its adoption of a liberalized economic model has generated the rapid industrialization of the country’s major cities. The country has attracted a large number of foreign investors to open businesses and factories in what are referred to as industrial zones (IZ) that support electronics, textiles, footwear, and automobile parts industries. To meet this labor demand, hundreds of thousands of women migrate from rural communities to cities in Vietnam to work in these IZs. Vietnam currently has 257 operational IZs, and they are staffed by approximately 3.6 million workers; a majority of these workers identify as women [28]. These migrant workers predominantly come from low-income, patriarchal families where they have typically had limited formal education, opportunities, and mobility [29,30]. Beyond these basic demographic characteristics, however, significant knowledge gaps persist in understanding these workers’ adaptations to and perceptions of their lives away from their families.

One of the numerous challenges that many women face in the IZs revolves around sexual and reproductive health (SRH) and HIV specifically. In Vietnam, SRH services mainly target married couples; consequently, unmarried women have limited access to SRH services [31]. While a large number of unmarried women migrant workers labor in IZs, there are currently no official government programs providing SRH services for them [32]. Due to the ho khau system policy [33], migrant women workers can only access publicly subsidized SRH/HIV services in their rural communities of origin. This means that women who have migrated to find work can only access healthcare services if they have the financial means to pay for health services in the city [31]. Consequently, the vast majority of women working in IZs only seek health care and SRH/HIV services in cases of extreme need. Limited studies in Vietnam suggest that women who are migrant workers in IZs are generally less formally educated than male migrant workers, lack access to SRH-related information, and are often at risk for domestic violence, non-consensual and/or unsafe sex, and sex work [32,34].

While a recent study among IZ migrant workers reported a lack of accurate knowledge about HIV and STIs [35], there are no studies that specifically investigate the prevalence of stigmatizing attitudes and behaviors toward PLWH among this type of at-risk population in IZs in Vietnam. What contemporary research does exist around stigma prevalence and persistence in Vietnam focuses on other demographic groups. Important work is being performed to understand stigmatizing experiences at both the personal and socio-cultural level, but such work primarily or exclusively focuses on critically impacted or at-risk groups such as MSM, individuals identifying as transgender, and pregnant women [36,37,38,39,40]. A few studies examine stigma in Vietnamese communities, but they do so within the context of the interactions between HIV status, alcohol use, and/or substance use disorders [41,42,43]. Lastly, a few notable studies took a geographic-based approach to mapping stigma, focusing on stigma in rural Vietnam [44,45]. As they relate to the current research, the findings from all these studies agree that HIV stigma is a persistent if not increasing challenge in numerous facets of Vietnamese society (i.e., interpersonal, healthcare, government policy, etc.) and within many different communities. They indicate a shared consensus that there continues to be a significant need to better map and understand the influences, drivers, and perpetuation of stigma among the many communities and regions of Vietnam. Such concerns are also echoed by relevant stigma assessment work being performed in countries such as China and Laos; their findings regarding the persistence of HIV stigma and possible interventions may have limited applicability to this topic as well [46,47,48]. Studies more than fifteen years old have not been considered in summarizing relevant research related to this topic. As outlined earlier in this introduction, this contemporary focus is needed due to the Vietnamese government‘s changes in HIV prevention and care, as well as the evolving discourse and tension among views regarding HIV and PLWH.

Existing research is clear that stigma remains a serious issue among Vietnamese communities and that this both impacts the quality of life of PLWH and creates barriers impacting the entire HIV care continuum. However, there are critical gaps in our understanding of the extent and pervasiveness of the influence of HIV stigma in underserved and often under-resourced working communities that are also vulnerable to HIV infection. The purpose of this study is to address this knowledge gap by quantifying the prevalence of stigmatizing attitudes regarding HIV and identifying correlative views that impact perceptions of PLWH among women migrant workers working in the industrial zones in Hanoi, Vietnam. Research by Pharris et al. suggests that young women living in rural areas are likely to have been exposed to stigmatizing perceptions regarding HIV [49], but there are no studies examining the interactions between stigmatizing views and working and living in IZs. The recent research summarized above also points to SRH as a possible mitigating factor against HIV stigmatization, but there are well-known limitations around access to HIV prevention services in IZs. Accordingly, we hypothesized that a majority of young women migrant workers would hold stigmatizing attitudes toward PLWH. We further hypothesized that young women migrant workers who did access SRH/HIV services while living in the IZ would be more likely to express less-stigmatizing attitudes toward PLWH than those who have not used such services. Addressing these two hypotheses will contribute to both public health and the sociological understanding of factors influencing HIV stigma—and will consequently contribute to policies and interventions to combat it—among this previously unstudied demographic in Vietnam.

## 2. Materials and Methods

### 2.1. Study Site and Participants

Data for this study were collected from a study project titled “HIV risk among young women migrant workers in the industrial zones in Vietnam” [50]. A cross-sectional study was conducted among 1061 young migrant women from January 2019 to November 2020 in Thang Long Industrial Park on the outskirts of Hanoi, the capital of Vietnam. The Park currently has 31 major industries, focusing on the assembly of consumer goods, electronic components, vehicle accessories, automotive electronics, and civil mechanical appliances [50]. The majority of the young women who work in IZs live in “rent clusters” that are privately owned and developed by landlords to accommodate the flow of workers seeking residences in the areas surrounding the Park. A minority of workers, particularly those who are just beginning their work in the IZ, live in dormitories provided by their factories.

Eligibility criteria for participation in the study involved self-identifying as: (1) a woman; (2) between 18 and 29 years of age; (3) either single, currently married but not living with a husband or partner while working in the IZ, separated, divorced, or widowed; (4) having worked in the IZ for six or more months; (5) from a rural area or another province prior to IZ employment. We chose women migrant workers only for this study because Vietnamese women in the IZs are particularly vulnerable to the risk of HIV transmission as they encounter male supervisors and male peers at work, male landlords and shopkeepers, and men in the community who seek their disposable income and sexual availability. These risks occur in a societal context of gender inequality, limited social supports, patriarchal norms, and job insecurity, as we note in our introduction.

The study was approved by the University of Connecticut Health Center Institutional Review Board and the Institute for Social and Medical Studies, Vietnam. Written informed consent in Vietnamese was obtained from all participants in the study.

### 2.2. Sample Size and Sampling Recruitment Process

Our study sample was assembled based on cluster sampling. Cluster sample designs that include people living/working in close proximity (such as women living together in a rent cluster or dormitory) can affect the power of a study due to the interactive effect of similarly behaving subjects. Our sample size determination accounted for the cluster design through intra-class correlation (ICC) within the primary sampling units: rent clusters and dormitories. We accounted for ICC by basing power on the effective sample size, according to according to Killip et al. [51], which resulted in a total number of 1061 participants required for the interviews.

Based on the desired sample size, our research team contacted the officials of the local administrative authority to obtain information about the rent clusters; the names of the owners, the total number of workers, and the number of women workers aged 18–29 in the residence were recorded. A multistage clustered sampling method was then used to enroll eligible participants. First, 779 rent clusters and two dormitories located near the IZ were mapped and enumerated. For the rent clusters, only rent clusters with six or more eligible participants were selected, resulting in the exclusion of 360 rent clusters and leaving 419 qualifying rent clusters. Once eligible rent clusters and dormitories were identified, women in the eligible age group were listed and contacted directly for screening and participation by the research team and collaborators who were former IZ women workers. A total of 1316 eligible migrant workers in the 419 rent clusters were selected to participate in the study; however, 320 of them refused participation, resulting in a total of 936 participants in the rent clusters being selected for interviews. For the two qualifying dormitories, 175 eligible participants from the list provided by the dormitory manager were contacted, but only 125 participants agreed to join the study.

### 2.3. Survey Interview

Eligible participants were invited to an after-hours meeting in a nearby commune health center organized by the research team in collaboration with guides who were current and former IZ women workers. At the meeting, they were fully informed about the study, the activities in which they were expected to participate, the topics to be covered by the survey, the amount of time required for the interview, their right to refuse or to withdraw, and the procedures for ensuring the confidentiality of interviews. A research team member then handed the participant a written informed consent form and asked her to read it. Any questions a participant had were answered by the research team members. When there were no further questions, the participant was given time to consider the risks and benefits of participation. Once agreeing to join the study, she was asked for written informed consent, after which she was enrolled. If they agreed to participate in the study, they were asked to sign written informed consent before participating in a face-to-face anonymous interview using closed-ended questions conducted by trained field researchers. The interview lasted for an hour. All interviewers received training in interviewing techniques, developing rapport, ensuring confidentiality, and answering questions raised by participants.

### 2.4. Measurement

#### 2.4.1. Socio-Demographic Variables

Recorded socio-demographic variables include age, marital status, education, ethnicity, length of employment in the IZ, housing status, working hours, and income.

#### 2.4.2. Assessing Stigmatizing Attitudes

A 4-item list of stigmatizing attitudes toward PLWH was used to capture negative perceptions. The measure was originally developed by the CDC [52] and was previously used in the National Survey on Sexual and Reproductive Health among Vietnamese Adolescents and Young Adults aged 10–24 [53]. The questions were: (1) “If a member of your family got infected with HIV, would you want to try to keep it secret?”; (2) “If a family member/relative of yours became sick with AIDS, would you be willing to care for him/her in your own household?”; (3) “If a worker was infected with HIV/AIDS, should he/she be allowed to continue working?”; and (4) “Would you buy food from a shopkeeper who was infected HIV/AIDS?” The closed response choices were “Yes,” “No,” and “Do not know.” Each question response was coded so that a non-zero value indicated a discriminatory attitude for all questions. Participants with an affirmative response to question one or negative responses to questions two through four were coded as having stigmatizing attitudes toward PLWH. The final composite score of stigmatizing attitudes ranged from 0 to 4, with a higher score indicating higher levels of stigmatizing attitudes toward PLWH.

#### 2.4.3. HIV/AIDS Knowledge

Knowledge of HIV/AIDS was measured with 7 yes/no/do not know questions [53]. Participants were asked a series of questions, including one item on HIV transmission (“HIV can be transmitted via unprotected sexual intercourse”), three items on the prevention of HIV (e.g., “people can protect themselves from HIV infection by using a condom correctly every time they have sex”), and three items about common HIV misconceptions. These last questions included statements about transmission via mosquito bites, transmission via sharing food with an individual living with HIV, and whether or not a healthy-looking person can have HIV/AIDS. Each correct answer was given one point, with a total score ranging from 0 to 7 points. A higher score indicated greater HIV knowledge.

#### 2.4.4. Utilization of SRH/HIV Services

Participants were asked if they had used sexual and reproductive health (SRH) or HIV services since they had begun working in the IZ. If yes, they were then asked to respond yes or no to each item on a standardized list of SRH and HIV services (e.g., HIV test, pregnancy care and/or delivery, gynecological exam, etc.).

#### 2.4.5. Data Analysis

Univariate analysis was conducted to obtain descriptive statistics of all variables. Frequencies and percentages were computed for categorical variables and as means for continuous variables. Chi-square was used to examine the associations between stigmatizing attitudes and socio-demographic characteristics. Multiple logistic regression analysis was used to examine factors associated with stigmatizing attitudes while controlling for demographic factors (age, education, marital status, ethnicity, and place of residence). Missing data were imputed using the median because the distribution of variables was not normally distributed. We computed the median using non-missing values of the variables of interest (e.g., stigmatizing attitudes and knowledge of HIV/AIDS) and used it to impute missing values. Nagelkerke R^2^ and χ^2^ were used to explain the model and effect sizes. A *p*-value was considered significant at <0.05.

## 3. Results

### 3.1. Socio-Demographic Characteristics

A total of 1061 young women workers were recruited and interviewed. The mean age was 22.8 years (ranging from 18 to 29, standard deviation = 2.8) (Table 1). The majority of respondents (79.4%) had completed high school. Over two-thirds of the respondents (*n* = 760) were single/unmarried (71.6%). Mean income was USD 294 per month, ranging from USD 195 to USD 652.

### 3.2. Stigmatizing Attitudes toward PLWH

Over seventy-six percent (76.2%) of the respondents reported having at least one of the four stigmatizing attitudes (Table 2). Considering each item individually, the most prevalent stigmatizing attitude was a negative response to “I am willing to buy foods from a shopkeeper with HIV/AIDS” (53.5%), followed by 47.4% of participants disagreeing with the statement that “a worker with HIV should be allowed to continue working”.

Conversely, 27.2% reported that they “would try to keep it secret if a member of your family got infected with HIV” and only 8.3% of respondents indicated that they would not be willing to care for a relative with HIV/AIDS at home.

### 3.3. Socio-Demographic Factors and Stigmatizing Attitudes toward PLWH among Respondents

Table 3 presents the results of the socio-demographic characteristics of the participants in relation to stigmatizing attitudes. Stigmatizing attitude was dichotomized into “having stigmatizing attitude” (those who responded to at least one of four questions in a way that demonstrated stigmatizing attitudes) and “having no stigmatizing attitude” (those who did not endorse stigmatizing attitudes in response to any question). Participants with less than a high school education reported higher levels of stigmatizing attitudes than participants who completed high school and above (*p* = 0.035). Participants who belonged to Kinh (the ethnic majority in Vietnam) were more likely to report having stigmatizing attitudes than those who belonged to ethnic minority groups (*p* = 0.015).

### 3.4. Multiple Stepwise Regression Analysis

Table 4 presents the multiple logistic regression analysis results. These results indicate that those with lower measures of HIV knowledge were more likely to express greater stigmatizing attitudes toward PLWH (*p* < 0.001). The overall model is statistically significant: χ^2^ (9) = 47.06, *p* < 0.01. The model explained 9% of the variance in stigma attitudes toward PLWH (Nagelkerke R^2^ = 0.09) and indicated a small effect size. Education level was also moderately correlated with stigmatizing attitudes (*p* < 0.01). Contrary to our secondary hypothesis, no statistically significant differences were found among participants who had ever used SRH/HIV services and those who had not.

Interaction effects between predictor variables and possible confounding factors (marital status, age, education, and residence) in the regression model were also assessed. The results showed that the interaction effects between HIV knowledge and factors including marital status, age, education, and residence on stigmatizing attitudes were nonsignificant: marital status (aOR = 0.96, 95% CI = 0.69–1.33, *p* = 0.805), education (aOR = 1.56, 95% CI = 0.80–3.01, *p* = 0.190), age (aOR = 0.89, 95% CI = 0.65–1.23, *p* = 0.492), and residence (aOR = 1.76, 95% CI = 0.89–3.77, *p* = 0.074). In addition, there were no interaction effects observed between the use of SRH/HIV and marital status (aOR = 2.26, 95% CI = 0.97–4.60, *p* = 0.065), age (aOR = 1.36, 95% CI = 0.71–2.63, *p* = 0.358), education (aOR = 0.95, 95% CI = 0.29–2.99, *p* = 0.92), or residence (aOR = 0.44, 95% CI = 0.17–1.60, *p* = 0.098) on stigmatizing attitudes.

## 4. Discussion

### 4.1. Prevalence of Stigmatizing Attitudes

This is the first known study to directly examine the prevalence of stigma toward PLWH among women migrant workers working in the industrial zones in Vietnam. Consistent with our hypothesis, the study findings demonstrated that a high proportion of women migrant workers have at least some stigmatizing attitudes toward PLWH; over seventy-six percent (76.2%) of respondents reported having at least one of the four measures of stigmatizing attitudes. This suggests that stigmatizing attitudes toward PLWH in Vietnam remain prevalent, at least among this demographic group. These results are consistent with similar findings regarding stigmatizing attitudes toward PLWH among migrant workers in China [47,54] and Nepal [55]. It is also in line with findings from a 2008 study by Hong et al. that found that rural-to-urban migrant workers in China possessed high measures of stigmatizing attitudes toward PLWH [56]. This current study’s data suggest a number of possible factors that may explain the differing levels of stigmatizing attitudes toward PLWH among study participants.

First, the majority of these migrant workers were from rural areas with limited access to educational opportunities generally and HIV/SRH educational programs specifically. In our study, education appeared to be a dominant predictive factor for expressions of stigma; participants with lower levels of HIV knowledge were more likely to respond with a higher measure of stigmatizing attitudes toward PLWH. Further, those respondents with lower overall education levels were more likely to express higher rates of stigmatizing attitudes toward PLWH than those with a high school education (more than ten years of schooling) and above. These correlational data with both education generally and HIV knowledge specifically seem to influence respondents’ perceptions of HIV, interacting with the backdrop of existing historically and culturally based stigmatizing attitudes toward PLWH that are known to be prevalent in the rural provinces from which respondents migrated [49]. However, the inverse of this inference is equally true; higher scores in these two educational categories were associated with fewer culturally informed stigmatized perceptions of PLWH. The apparent mitigating effect of education on stigma among this population has significant implications for any possible stigma-reduction efforts in IZs and among young working women.

Secondly, while living in the IZ, these migrants typically have limited access to HIV education programs and prevention services. A 2012 study among IZ women migrant workers in Vietnam reported that only 21.6% of participants ever sought treatment from healthcare services when they suffered from reproductive tract infections [57]; it may well be that these low rates of SRH-related healthcare uptake also apply to other SRH needs, including those related to HIV or HIV exposure. Service uptake is further negatively impacted by the Vietnamese federal policy that states that migrant workers are not considered permanent residents in the city. As a result, they cannot be categorized as official target demographics for HIV education activities conducted by government public health agencies [31]. These factors contribute to what we have documented here, which is highly limited access to HIV information. This finding suggests that addressing stigmatizing attitudes toward PLWH and improving access to HIV education programs among this population require both local and national government agencies to develop policies and programs specifically targeting migrant workers. Such policy interventions would ensure that they have equitable access to healthcare and SRH/HIV services as local residents.

### 4.2. HIV Knowledge and Stigmatizing Attitudes

This study’s finding that participants with lower levels of HIV knowledge were more likely to report greater levels of stigmatizing attitudes toward PWH echoes similar findings reported among migrant workers in China [34] and other populations in both China [35] and Laos [36]. In summarizing the lessons learned from those research programs, Pulerwitz et al. (2010) reported that providing participants with accurate knowledge about HIV transmission can result in less fear and avoidance with respect to attitudes toward PLWH [58]. This similar work from nearby countries combines with our findings here to suggest that improving HIV knowledge among migrant workers in Vietnamese IZ settings may help reduce stigmatizing attitudes toward PLWH.

### 4.3. Use of SRH/HIV Services and Stigmatizing Attitudes

A noteworthy finding from this study is that women migrant workers who had used SRH/HIV services while living in the IZ did not demonstrate less-stigmatized attitudes toward PLWH in a statistically significant way. This disproves our hypothesis that accessing such services would mitigate stigmatizing attitudes. Our survey hypothesized that those who accessed these services might receive HIV-related counseling and/or HIV education that would decrease the fear of HIV and stigmatized attitudes toward PLWH. However, the study results did not find any significant differences between participants who had accessed SRH/HIV services and those who had not. One possible explanation for this finding could be that those services did not, in fact, dispense counseling or education related to HIV. While the exact underlying cause related to interactions in or with SRH settings is outside the scope of this study, these findings do highlight an important potential healthcare gap, an opportunity for targeted interventions, and a topic where further research is needed to more fully explore this issue.

While recognizing the work that has been performed around HIV education and prevention efforts at the community level [21] and the incorporation of HIV education into school settings across Vietnam in the past two decades [24], our research shows that stigma toward PLWH remains high in the study population. These high levels suggest that additional outreach activities and culturally appropriate interventions are needed to address stigmatizing attitudes toward PLWH among women migrant workers in IZs. Recent research suggests that interventions using mHealth (the use of mobile technologies to improve access to health information and health service delivery) may be an effective approach to reducing stigmatizing attitudes toward PLWH among this population. For example, this approach has been shown to be effective at increasing IZ women migrant workers’ general SRH knowledge in Vietnam [59], improving young people’s access to SRH services in LMICs [60], and reducing HIV-related stigma among youth in countries such as the United States [61].

### 4.4. Stigmatizing Attitudes and Role of Family

Finally, it is worth noting that the questions to which participants provided the most stigmatized responses were those that reference people outside of the respondent’s family. Nearly 92% of the participants indicated that they would be willing to care for a family member who became HIV positive. Statistically, most traditional Vietnamese family values are impacted by Confucianism, which teaches that taking care of family members when they are sick is very important [62]. In this traditional cultural view, women, in particular, are expected to stand by and support their husbands through illness [21]. Khuat (2004) documented this cultural norm, noting that mothers and wives try to find ways to make their sons/husbands feel comfortable when ill, including if they became sick because of HIV [23]. Our finding that the vast majority of respondents expressly stated their willingness to care for a family member who has HIV/AIDS has profound potential to influence further stigma-reduction efforts. This finding builds upon a small qualitative study conducted by Dinh et al. that showed complex interactions among family members navigating HIV diagnoses and disclosure and explored the mitigating role that strong family ties can play in associated stigma [45]. Our findings present the compelling suggestion that leveraging narratives relating to the importance of family and culturally appropriate messaging on the same topic may be a novel/underutilized narrative entry point for interventions and/or campaigns that seek to dismantle HIV-related stigma in Vietnam generally and among women in particular. In this way, the role of family members may be crucial not only in providing care to PLWH in Vietnam but also in significantly contributing to future stigma-reduction efforts.

While these findings on their own cannot be generalized to women in other communities in Vietnam, they do align with and offer more statistically powerful support for previous smaller studies involving women’s attitudes in other settings, such as Dinh et al. and Thi et al. These researchers’ populations (*n* = 12 and *n* = 53, respectively) offered evidence of stigmatizing attitudes among and toward the women in their qualitative or small sample studies [44,45]. Our more robust results more clearly demonstrate non-familial HIV stigma perpetuation among young women workers. These results also highlight the value of expanding stigma research into other settings and communities run or populated by women. One notable study conducting work along these lines is Oosterhoff and Bach’s 2013 study of HIV-positive pregnant women engaging in mutual support and grassroots group mobilization. Their results indicated some level of perpetuated stigma expressions and lower self-esteem experienced by the women in that study despite a number of other personal and social improvements [63]. Finally, our work with this specific working population suggests that longitudinal studies into women’s perceptions of HIV once they conclude their IZ work and enter other sectors of Vietnamese (or international) society would offer even greater insights into the perpetuation and dissemination of these views and, thus, expand and build on the results reported here.

### 4.5. Limitations

There are several limitations in this study. Given the nature of this cross-sectional design, our findings should not be interpreted beyond associations, and causality cannot be definitively demonstrated. Stigmatizing attitudes were measured using only four questions and did not comprehensively measure every domain of stigma or intersectional stigma. Generalizations of these findings cannot be made to other migrant populations or women in other settings and communities in Vietnam. Further, while the Thang Long Industrial Park is demographically similar to many of the IZs around Hanoi, caution must be exercised before assuming that its workers are necessarily representative of all IZs or in generalizing these findings across the many IZs in Vietnam. Finally, this study focused on recruiting women migrant workers and so any stigmatizing attitudes toward PLWH among men migrant workers, as well as perceptions among transgender individuals in Vietnam, may be different. In this regard, it is worth noting that gender identity is treated as a self-reported identifier throughout this study. Gender does not exist in a binary, and this study intentionally uses the broad and non-biologically deterministic term ‘women’ throughout to include all participants who identify as women regardless of their sex assigned at birth. Researching stigma and other experiences among transwomen and all gender expressions throughout Vietnamese communities is beyond the scope of this study but would be a fruitful and important avenue for future research.

## 5. Conclusions

This research builds on and expands recent past research around HIV stigma in Vietnam by including Vietnam’s understudied populations living in IZs, specifically, young migrant women workers. The study shows both widespread and high levels of stigmatizing attitudes toward PLWH among this population in the IZ study site outside Hanoi, Vietnam. Knowledge around the persistence of HIV stigma in Vietnam remains incomplete, but stigma is well-known to impact both quality of life for PLWH and the care continuum in Vietnam. Our work here addresses an expansion of this knowledge base and offers a critical juncture to identify and impact this issue. First, it provides novel and much-needed additional data supporting the prioritization of HIV education intervention programs generally and the need for efforts to reduce stigma toward PLWH among migrant women working in IZs in Vietnam specifically. Our data indicate that more widespread implementation or support for education in rural areas and/or SRH and HIV education in IZs would, beyond their obvious primary benefits impacting education, public health, and quality of life for young women, also likely be associated with decreased levels of HIV-related stigma and stigma against PLWH. Secondly, it provides critical evidence and data-based suggestions for policymakers and healthcare leaders regarding improvements to conditions and policies around SHR and HIV, as well as the need to improve access to HIV prevention and treatment services among migrant workers working in IZs in Vietnam. In this regard, the current study adds to both the existing literature and current efforts and policies around HIV in Vietnam by empirically suggesting that familial-based messaging could be a powerful potential intervention narrative for addressing HIV-related issues such as stigma.

## Figures and Tables

**Table 1:** Participant characteristics.

Characteristics	Total (*n* = 1061)	
	*n*	% or Mean (SD)
Age (years)	1061	22.8 (3.3)
18–24	733	69.1
25–29	328	30.9
Education		
5–9 years	109	10.3
10–12 years	842	79.4
≥12 years	110	10.4
Ethnicity		
Kinh (majority group)	735	69.3
Other (minority groups)	326	30.7
Marital status		
Unmarried	760	71.6
Married *	301	28.4
Residence		
Dormitory	125	11.8
Rent cluster	936	88.2
Years of working at the current IZ		
≤1 year	287	27.1
>1–5 years	625	58.9
>5 years	149	14.0
Daily shift working hours		
≤8 h/day	810	76.3
Over 8 h/day	251	23.7
Monthly income		
mean	1061	USD 294.0
min		USD 195.0
max		USD 652.2

* Married includes those who were married and not living with their husbands and those who were separated, divorced, or widowed.

**Table 2:** Stigmatizing attitudes toward PLWH among women migrant workers (*n* = 1061).

Stigmatizing Attitudes	*n* (%)
If a member of your family got infected with HIV, would you want to try to keep it secret? (agreed)	288 (27.2)
If a member/relative of yours became sick with AIDS, would you be willing to care for him/her in your own household? (disagreed)	88 (8.3)
If a worker was infected with HIV/AIDS, should he/she be allowed to continue working? (disagreed)	502 (47.3)
Would you buy food from a shopkeeper who was infected HIV/AIDS? (disagreed)	567 (53.5)
Had at least one stigmatizing attitude	809 (76.2)

**Table 3:** Stigmatizing attitudes by demographic characteristics among those who possessed stigmatizing attitudes toward PLWH.

Characteristics	Stigmatizing Attitudes *		
	*n*	Percent	*p*
Age			
18–24	530	75.8	0.354
25–29	279	77.1	
Education			
5–9 years	94	86.2	
10–12 years	632	75.1	0.035
≥12 years	83	75.5	
Marriage			
Unmarried	583	76.7	
Married	226	75.1	0.576
Ethnicity			
Other (minority)	233	71.5	
Kinh (the majority)	576	78.4	0.015
Residence			
Dormitory	94	75.4	0.768
Rent cluster	715	76.4	
Monthly income			
Low	471	77.6	0.234
High	338	74.4	
Used SRH/HIV services while living in the IZ			
No	536	77.3	0.141
Yes	273	74.2	
Ever taken an HIV test			
No	675	77.1	0.084
Yes	134	72.0	

* Stigmatizing attitude was dichotomized into “having stigmatizing attitude” (those who responded to at least one of four questions in a way that demonstrated stigmatizing attitudes) and “having no stigmatizing attitude” (those who did not endorse stigmatizing attitudes in response to any question).

**Table 4:** Factors associated with stigmatizing attitudes toward PLWH among women migrant workers.

Characteristics	aOR	95% CI	*p*
Age			
18–24	1		
25–29	1.40	0.96–2.05	0.076
Education			
5–9 years	1		
≥10 years	0.44	0.24–0.80	0.007
Marital status			
Single	1		
Ever married	0.85	0.55–1.29	0.454
Ethnicity			
Other (minority) group	1		
Kinh (the majority) group	1.39	1.02–1.89	0.036
Residence type			
Dormitory	1		
Rent cluster	1.08	0.68–1.71	0.730
Knowledge of HIV/AIDS	0.69	0.60–0.80	<0.001
Used SRH/HIV ^a^ services while living in the IZ ^b^			
No	1		
Yes	0.85	0.60–1.21	0.375
Ever taken an HIV test			
No	1		
Yes	0.83	0.57–1.21	0.228

Note: The overall model is statistically significant: χ^2^ (9) =47.06, *p* < 0.01; Nagelkerke R^2^ = 0.09. aOR: adjusted Odd Ratio. CI: confidence interval. ^a^ SRH/HIV: sexual and reproductive health/HIV; ^b^ IZ: industrial zones.

## Data Availability

The data presented in this study are available on request from the corresponding author.
